# ID 111 - Curso Teórico-Prático de Busca Avançada na Literatura para Identificação de Evidências Científicas para Técnicos dos NatJus

**DOI:** 10.5327/2237-9622.2025.v34s1.111

**Published:** 2025-11-25

**Authors:** Carolina de Oliveira Cruz Latorraca, Isabela Porto de Toledo, Rafael Leite Pacheco, Verônica Colpani, Ana Luiza Cabrera Martimbianco, Camila Monteiro Cruz, Cecília Menezes Farinasso, Patrícia do Carmo Silva Parreira, Rachel Riera

**Keywords:** judicialização da saúde, base de dados, cursos de capacitação, avaliação de tecnologia biomédica

## Abstract

**Introdução:**

Notas técnicas (NTs) são documentos que respondem demandas judiciais específicas apresentando evidências científicas para uma tomada de decisão judicial mais bem informada. As NTs são elaboradas por profissionais da saúde atuantes nos Núcleos de Apoio Técnico ao Judiciário (NatJus). Os métodos para busca de estudos e identificação de evidências científicas na literatura se renovam continuamente e é fundamental que as competências dos elaboradores de NT sejam constantemente atualizadas para assegurar qualidade, transparência e imparcialidade. O objetivo deste estudo foi relatar a realização e os resultados de um curso teórico-prático de capacitação em estratégias de busca complexas para membros dos NatJus do Brasil elaboradores de NT ou parceiros dos Tribunais de Justiça que exerçam esta atividade e técnicos do Ministério da Saúde (MS) indicados pelo Departamento de Gestão das Demandas em Judicialização na Saúde (Djud).

**Método:**

Estudo transversal conduzido pelo Núcleo de Avaliação de Tecnologias em Saúde/Núcleo de Evidências, Hospital Sírio-Libanês (HSL), São Paulo, Brasil, como uma entrega do projeto "AD-Jus: Apoio técnico-científico à tomada de decisão judicial em Saúde no Brasil", desenvolvido pelo HSL e apoiado pelo MS por meio do Programa de Apoio ao Desenvolvimento Institucional do Sistema Único de Saúde (Proadi-SUS). O curso teve como objetivo contribuir com a aquisição de conhecimento e desenvolvimento de competências relacionadas a buscas estruturadas em bases de dados científicas nacionais e internacionais, gerais e específicas, e buscas manuais em websites institucionais e outras fontes relevantes para identificação de estudos que podem compor o conjunto de evidências de uma NT. Foi realizada uma atividade pré/pós-curso para avaliação da aquisição do conhecimento e avaliação de satisfação dos participantes.

**Resultados:**

Este curso teórico-prático de curta duração e de inscrição gratuita focou no aprofundamento da elaboração de estratégias de busca complexas para as principais bases de dados da saúde de acesso gratuito. Foi composto por dois dias de atividades teórico-práticas remotas síncronas com duração de oito horas/dia (16 horas) com a realização de estudo dirigido, exercícios práticos, discussões e entrega de produto final individual. Foram 77 participantes inscritos e 55 aprovados para certificação.

Foi observada uma média de satisfação geral alta (>75%) a partir dos questionários de satisfação aplicados. A satisfação quanto ao conteúdo do curso foi considerada ótima (88,5%), quanto aos recursos foi mediana a alta (50 a 70%), com os professores apresentou uma média alta (>75%) e 83% dos respondentes referiram que é extremamente provável a recomendação do curso para outra pessoa.

Quanto à avaliação pré/pós-curso, seis das sete questões tiveram aumento da frequência de respostas corretas. O aumento na porcentagem de acertos entre as questões avaliativas foi, em média, de 15,2% após o curso, quando comparado às respostas pré-curso.

**Conclusão:**

O cenário geral aponta para uma satisfação alta, aumento da frequência de acertos entre os participantes no pós-curso e a recomendação para outra pessoa é extremamente provável. As atividades práticas, o material utilizado, a didática dos professores e a distribuição em pequenos grupos para tutorias podem ter sido relevantes para esse aumento.

